# Taq1A polymorphism and medication effects on inhibitory action control in Parkinson disease

**DOI:** 10.1002/brb3.1008

**Published:** 2018-06-01

**Authors:** Katherine E. McDonell, Nelleke C. van Wouwe, Madaline B. Harrison, Scott A. Wylie, Daniel O. Claassen

**Affiliations:** ^1^ Department of Neurology Vanderbilt University Medical Center Nashville Tennessee; ^2^ Department of Neurology University of Virginia Charlottesville Virginia; ^3^ Department of Neurosurgery University of Louisville Louisville KY United States

**Keywords:** dopamine agonist, dopamine receptor, genetic polymorphism, impulse control disorder, Parkinson disease

## Abstract

**Background:**

Dopamine therapy in Parkinson disease (PD) can have differential effects on inhibitory action control, or the ability to inhibit reflexive or impulsive actions. Dopamine agonist (DAAg) medications, which preferentially target D2 and D3 receptors, can either improve or worsen control of impulsive actions in patients with PD. We have reported that the direction of this effect depends on baseline levels of performance on inhibitory control tasks. This observation suggests that there may exist certain biologic determinants that contribute to these patient‐specific differences. We hypothesized that one important factor might be functional polymorphisms in D2‐like receptor genes.

**Aim:**

The goal of this study was to determine whether the direction of DAAg effects on inhibitory control depends on functional polymorphisms in the DRD2 and DRD3 genes.

**Methods:**

Twenty‐eight patients with PD were genotyped for known functional polymorphisms in DRD2 (rs6277 and rs1800497) and DRD3 (rs6280) receptors. These patients then completed the Simon conflict task both on and off DAAg therapy in a counterbalanced manner.

**Results:**

We found that patients with the rs1800497 Taq1A (A1) polymorphism (A1/A1 or A1/A2: 11 subjects) showed improved proficiency to suppress impulsive actions when on DAAg; conversely, patients with the A2/A2 allele (14 patients) became less proficient at suppressing incorrect response information on DAAg therapy (Group × Medication, *F*(1, 23) = 5.65, *p* < 0.05). Polymorphisms in rs6277 and rs6280 were not associated with a differential medication response.

**Conclusion:**

These results suggest that certain DRD polymorphisms may determine the direction of DAAg effects on critical cognitive control processes impaired in PD. Our findings have implications for understanding pharmacogenomics interactions on a larger scale and the role these may play in the wide variability of treatment effects seen in the PD population.

## 1 INTRODUCTION

Dopaminergic medications remain the mainstay treatment for Parkinson disease (PD) motor symptoms, but a growing body of research reveals both beneficial and detrimental effects of dopaminergic medications on specific cognitive processes in PD. One influential theory asserts that the effect of dopaminergic medications depends largely on the baseline performance in an off medication state (Cools & D’Esposito, 2011). Patients with PD who show impaired performance in an off medication state, presumably due to greater DA depletion, typically benefit from the addition of dopaminergic medications, whereas individuals who show near‐normal performance in an off state often show decline in performance when dopaminergic medications are added (Cools, Barker, Sahakian, & Robbins, 2001; Wylie, Claassen et al., 2012). This inverted U‐shaped DA performance curve is suggestive of differential dopaminergic pathology in cognitive circuits across patients (Cools & D’Esposito, 2011).

Although differential progression of DA loss in key cognitive circuitries is a putative explanation for the dissociable medication effects on cognitive performance, genetic differences in the expression and function of DA receptors may also be a critical determinant in the trajectory of an individual’s response to DA medications, especially receptor agonists. An alternative, or at least a complementary factor, to the DA pathology view of performance is that individual differences in the genetics and resulting functional integrity of the DA system contribute to the baseline performance differences among patients with PD and their unique response to dopaminergic medications.

In a recent study, we reported a baseline‐dependent effect of DA agonist (DAAg) medication on a key component of the executive cognitive control system, the ability to inhibit reflexive or impulsive actions (Wylie, Claassen et al., 2012). A deficit in the proficiency of inhibiting impulsive actions has been reported across several studies of PD, mostly in the “on” DA state. Impairments in inhibitory action control are associated with greater postural instability, fall risk, and track positively with worsening disease severity in PD (Wylie, Ridderinkhof, Bashore, & van den Wildenberg, 2010; Wylie, van den Wildenberg et al., 2012). The network underlying inhibitory action control relies on a well‐described motor‐inhibitory circuit, which includes the ventrolateral prefrontal cortex, presupplementary motor cortex, and basal ganglia, with a particularly important role of the indirect and hyperdirect pathways engaging the subthalamic nucleus (STN) (Richard Ridderinkhof, Forstmann, Wylie, Burle, & van den Wildenberg, 2011). Decreased nigrostriatal DA in PD alters the proficiency of this network, which is putatively a key factor in driving the dysfunction in inhibitory action control (Wylie et al., 2010). We found that individuals who showed the largest impairments in inhibitory control off their DAAg medication experienced substantial improvements when taking their medication, whereas individuals who showed near‐normal inhibitory control off agonist experienced a substantial disruption to the proficiency of inhibitory when taking their medication (Wylie, Claassen et al., 2012). This observation provides direct support for the modulatory role of DA in inhibitory control and the crucial role of baseline performance. This differential medication effect, contingent on baseline levels of inhibitory control, raises an important question regarding individual differences in response to medication. That is, are there biologic determinants of medication responsiveness in PD?

In the current investigation, we obtained genetic polymorphism data for DA receptor genes in a subset of patients who completed the prior study of inhibitory control. We hypothesized that functional polymorphisms in DA receptor genes may contribute to patient‐specific differences in baseline inhibitory control performance and the response to DAAg therapy. DA agonists (commonly prescribed as ropinirole, pramipexole, or rotigotine) preferentially target D2‐like receptors (specifically the *DRD2* and *DRD3* receptors), which are predominantly expressed in the mesolimbic, mesocortical, and indirect basal ganglia pathway (Albin, Young, & Penney, 1989) (Gerfen et al., 1990). The goal of this study was to determine whether the direction of DAAg effects on inhibitory control depends on functional polymorphisms in the *DRD2* and *DRD3* genes. A number of polymorphisms in these genes have been associated with impulse control disorders such as addiction and excessive reward‐driven behaviors (as described below), suggesting some possible links to inhibitory control dysfunction.

### 1.1 Genetic polymorphisms affecting dopamine receptor genes

Three polymorphisms were included in this analysis: the rs6277 and rs1800497 polymorphisms in the *DRD2* gene and the rs6280 polymorphism in the *DRD3* gene. D2‐like receptors are expressed in the midbrain as well as throughout the dorsal and ventral striatum. They are localized both pre‐ and postsynaptically and primarily act to modulate and inhibit DA transmission (Baik, 2013). The D3 receptor is predominantly expressed in the ventral striatum and is thought to act as a presynaptic autoreceptor, inhibiting dopamine release (Bouthenet et al., 1991; Diaz et al., 2000). It is also found more broadly in the substantia nigra, hypothalamus, globus pallidus, and thalamus (Rabiner et al., 2009; Tziortzi et al., 2011).

The Taq1A polymorphism (rs1800497), representing the A1 allele of the DRD2/ANKK1 gene, has been associated with disorders of self‐regulation, such as obesity (Comings et al., 1993; Wang et al., 2001), addiction (Berggren et al., 2006; Blum et al., 1990), and impaired executive function (Ariza et al., 2012). The presence of the A1 allele is functionally related to lower D2 receptor striatal density and DA substrate‐binding specificity. Studies have shown that the D2 receptor density can be reduced by up to 30% in A1 carriers, particularly in the ventral regions of the caudate and putamen (Jonsson et al., 1999; Pohjalainen et al., 1998; Ritchie & Noble, 1996). This allele has also been associated with reduced glucose metabolism in the striatum as well as the ventral and medial prefrontal cortex (Noble, Gottschalk, Fallon, Ritchie, & Wu, 1997).

The rs6277 polymorphism in the *DRD2* gene is another important variant that is thought to regulate D2 receptor availability in the striatum. The homozygous T/T genotype is associated with the greatest receptor availability, followed by T/C, and then C/C, which is associated with the lowest availability. This is supported by PET imaging studies showing lower striatal DRD2‐binding potential in individuals with the C allele than in those with the T allele (Hirvonen et al., 2004). Low DRD2 receptor density associated with the C allele has also been identified as a risk factor for addictive behaviors such as alcoholism (Repo et al., 1999; Swagell et al., 2012) and tobacco abuse (Perkins et al., 2008; Voisey et al., 2012).

The third polymorphism included in our analysis was the rs6280 (Ser9Gly) polymorphism in the DRD3 gene. The Gly allele (C/C or C/T) increases DA affinity for the D3 receptor. This increased affinity is thought to lead to greater reward‐related DA release, primarily through an increase in phasic DA signaling (Savitz et al., 2013). The Ser9Gly polymorphism has been associated with a variety of substance abuse (Agrawal et al., 2013; Kuo et al., 2014; Novak et al., 2010; Vandenbergh et al., 2007; Wei et al., 2012) as well as mood disorders (Dikeos et al., 1999; Schosser et al., 2011). Animal studies have also shown that D3 receptor antagonists can reduce the likelihood of relapse into alcohol (Vengeliene et al., 2006) tobacco (Khaled et al., 2010) and cocaine‐seeking behaviors (Xi et al., 2004).

We hypothesized that the presence of polymorphisms previously associated with reduced behavioral control would be associated with a lower level of inhibitory control at baseline (i.e., A1 and C alleles in the DRD2 gene and the C allele in the DRD3 gene) and would show a greater improvement in measures of inhibitory control on DAAg therapy, while polymorphisms linked to better behavioral control would be associated with normal or higher level of inhibitory control at baseline and a negative response to DAAg therapy.

## 2 METHODS

Twenty‐eight patients with a diagnosis of idiopathic PD completed the Simon conflict task (described below) on and off DAAg therapy in a counterbalanced manner. All patients represent a subset of patients described in a previous report (Wylie, Claassen et al., 2012), but for whom genetic data were also collected at random. Seventeen of the 28 patients were taking levodopa along with a DAAg, and 11 were taking an agonist alone. DAAg doses were converted to levodopa equivalent daily dose (LEDD) values (Weintraub et al., 2006). For the off state, participants were withheld from DAAg for 24 hr. Patients were genotyped for known functional polymorphisms in *DRD2* (rs6277 and rs1800497) and *DRD3* (rs6280) receptors.

All participants were recruited and evaluated at the Movement Disorders Clinic. A neurologist specializing in movement disorders confirmed the diagnosis of idiopathic PD, and motor symptom severity was graded using the Unified Parkinson’s Disease Rating Scale (UPDRS) motor subscore obtained during each patient’s “on” medication state. Prior to entry into the study, patients’ medical histories were carefully reviewed, and they were screened for global dementia and major depression using the Mini‐Mental State Exam (Folstein, Folstein, & McHugh, 1975) and Center for Epidemiologic Studies Depression Scale (CES‐D; Radloff, 1977), respectively.

The Questionnaire for Impulsive‐Compulsive Disorders in Parkinson’s Disease (QUIP) was completed by each patient and by a spouse or reliable informant (Weintraub et al., 2009). This instrument screens for the presence or absence of any of the primary impulse control disorder (ICD) symptoms, including pathological gambling, compulsive buying, compulsive eating, hypersexuality, and for secondary manifestations such as compulsive hobbyism, punding, and DA dysregulation syndrome. Patients and their informants were also interviewed to confirm whether their behavior met established criteria for ICD behaviors (Voon, Kubu, Krack, Houeto, & Troster, 2006).

Patients were excluded if they had a history of comorbid neurological condition such as stroke, peripheral neuropathy, or seizure disorder; untreated or unstable mood disorder such as major depression; dementia; history of bipolar affective disorder, schizophrenia, or other psychiatric conditions known to compromise cognition; or untreated or unstable medical condition known to interfere with cognition such as diabetes or pulmonary disease. All participants had normal or corrected‐to‐normal vision. Prior to study entry, participants provided informed consent, which was compliant with standards of ethical conduct in human investigation.

### 2.1 Simon conflict task

The Simon task (Simon, 1967) measures an individual’s susceptibility to acting on strong action impulses and the proficiency of inhibiting these impulses as an act of cognitive control. The task’s elegance lies in its simplicity to administer, coupled with its elicitation of a highly robust conflict effect between impulsive action tendencies and desired actions. Participants view a series of colored circles presented one at a time to the left or to the right of a central fixation point on a computer screen. Participants issue a left or right‐hand button response based on a predetermined mapping between the color of a circle and a response hand (e.g., blue circle = left‐hand button press; orange circle = right‐hand button press). The spatial position of the circle to the left or right visual half‐field, although an irrelevant stimulus feature in terms of the task goal, elicits an automatic impulse to respond with the hand on the same side as the stimulus. On corresponding (Cs) trials, the response activated impulsively by the spatial position of the circle is the same response signaled by the circle’s color (e.g., a blue circle calling for a left‐hand response appears to the left visual hemifield). When the two responses correspond, performance is facilitated, as evidenced by faster reaction times (RT) and high accuracy rates. In contrast, on noncorresponding (Nc) trials, the response activated impulsively by the spatial position of the circle conflicts with the response signaled by the circle’s color (e.g., a blue circle calling for a left‐hand response appears to the right visual hemifield). When the two responses conflict, performance is compromised as RT slows and error rates increase. Simon effects are calculated as the average costs to RT and accuracy on Nc trials compared to Cs trials, which provide a measure of the magnitude of interference from conflicting response impulses, and inferentially, the extra time required to resolve this conflict.

### 2.2 Data analysis

We performed three sets of data analyses separately for each of the distinct genetic polymorphisms. First, we analyzed mean interference costs on RT and accuracy rates (square root‐transformed) between Nc and Cs trials (within‐subject factor, *Correspondence*). Next, we isolated impulse capture by analyzing patterns of fast impulsive action errors using accuracy rates from the fastest bin of the CAF. At last, we analyzed the proficiency of inhibiting these impulses using the slope reduction in the Simon interference effect between the final two bins of the delta plot. For each set of analyses, we included a within‐subject factor, *agonist state*, which consisted of two levels, on and off agonist medication. We also included a between‐subject factor indicating the presence or absence of a particular feature for each of the genetic polymorphism groups (presence vs. absence of A1 allele [Taq1A polymorphism] in *DRD2* gene, presence vs. absence of C allele [rs6277 polymorphism] in *DRD2* gene, and presence vs. absence of C allele [Ser9Gly polymorphism] in *DRD3* gene). Repeated measures analysis of variance techniques were used to analyze all data. Given that this is a re‐analysis of previously reported data, the reader is referred to our prior study for additional methodological and analytic details (Wylie, Claassen et al., 2012).

## 3 RESULTS

Tables 1, 2, 3 show the patient demographics in each genotype group. There were no significant differences between genotype subgroups in terms of disease duration, UPDRS score, or DA medication dosages in the *DRD2* groups. In the *DRD3* group, patients with the rs6280 polymorphism had slightly fewer years of formal education, were more likely to be male, and had a greater prevalence of ICD symptoms and higher UPDRS motor score than those without this polymorphism.

**Table 1:** Participant demographics by *DRD2* Taq1 rs1800497 group (average ± standard error of mean)

	AA/AG	GG	*p*‐value
Sample size	11	14	
Age	60.0 (1.9)	63.2 (1.9)	0.25
Education	17.8 (0.7)	17.1 (0.5)	0.46
Gender (M:F)	8:3	6:8	0.02
Depression rating	8.6 (1.4)	12.7 (2.4)	0.18
ICD (present:absent)	8:3	8:6	0.02
MMSE	29.3 (0.4)	29.1 (0.3)	0.77
UPDRS motor	17.5 (2.3)	15.9 (2.3)	0.61
Disease duration	6.3 (1.8)	7.9 (1.6)	0.53
Agonist equivalent	200.2 (34.1)	233.4 (33.0)	0.50

*Note*

ICD: impulse control disorder; UPDRS: Unified Parkinson’s Disease Rating Scale.

**Table 2:** Participant demographics by *DRD2* rs6277 group (average ± standard error of mean)

	CC/CT	TT	*p*‐value
Sample size	18	7	
Age	62 (1.7)	61.3 (2.5)	0.8
Education	17.2 (0.5)	18 (1.0)	0.4
Gender (M:F)	10:8	4:3	1.0
Depression rating	10.8 (1.6)	11.3 (3.7)	0.9
ICD (present:absent)	8:10	3:4	1.0
MMSE	29.2 (0.2)	29.2 (0.6)	0.8
UPDRS motor	15.9 (1.9)	18.4 (2.9)	0.5
Disease duration	7.0 (1.4)	7.6 (2.6)	0.8
Agonist equivalent	121.5 (28.6)	176.8 (39.2)	0.3

*Note*

ICD: impulse control disorder; UPDRS: Unified Parkinson’s Disease Rating Scale.

**Table 3:** Participant demographics by *DRD3* rs6280 group (average ± standard error of mean)

	CC/CT	TT	*p*‐value
Sample size	11	13	
Age	61.2 (2.0)	62.8 (2.0)	0.6
Education	16.4 (0.7)	18.6 (0.4)	0.007
Gender (M:F)	9:2	4:9	<0.0001
Depression rating	11.9 (1.8)	10.46 (2.5)	0.6
ICD (present:absent)	7:4	4:9	0.02
MMSE	29.1 (0.4)	29.3 (0.3)	0.6
UPDRS motor	20.4 (2.0)	13.4 (2.3)	0.035
Disease duration	9.0 (2.12)	5.8 (1.4)	0.2
Agonist equivalent	233.2 (39.8)	206.2 (31.4)	0.6

*Note*

ICD: impulse control disorder; UPDRS: Unified Parkinson’s Disease Rating Scale.

### 3.1 *DRD2* Taq1 rs1800497

#### 3.1.1 Mean RT and accuracy rates

**Figure 1:**
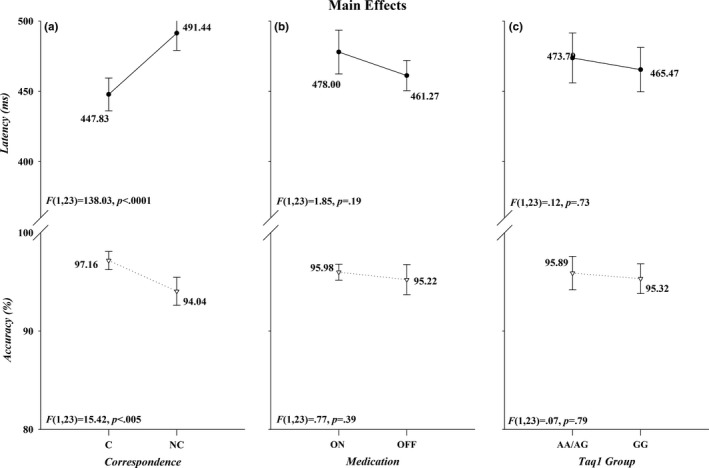
The effects of Correspondence (panel a), Medication (panel b), and Group (panel c) on RT (upper half of each panel) and accuracy (lower half of each panel). The *F* ratios and *p* values associated with each main effect are shown in the lower left of each half‐panel

**Figure 2:**
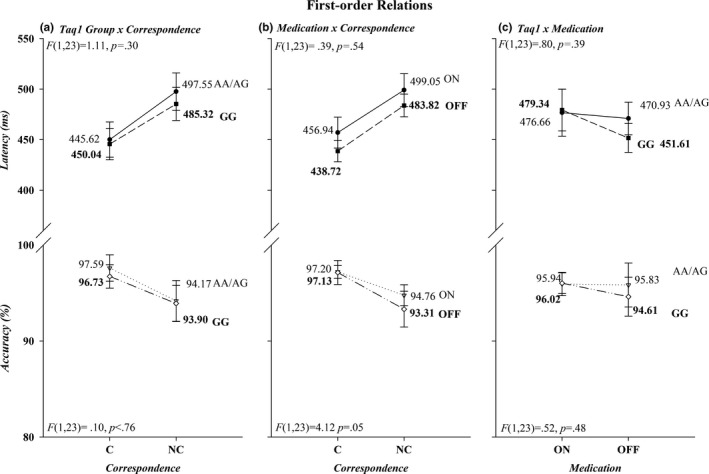
First‐order interactions are shown in panels (a), (b), and (c) for both RT (upper half‐panels) and accuracy (lower half‐panels). RT: reaction times

**Figure 3:**
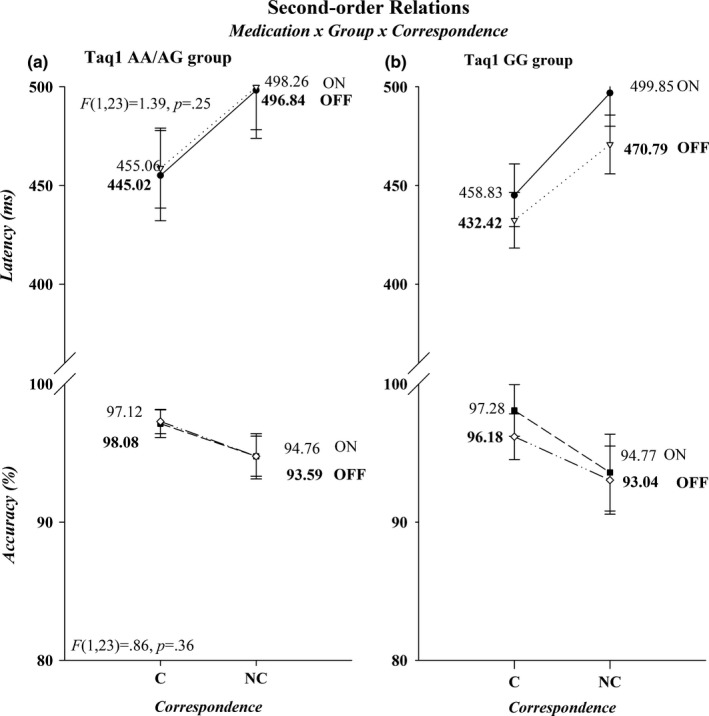
Second‐order interactions are depicted for both RT (upper half‐panels) and accuracy (lower half‐panels). The effects for Taq1A groups AA/AG and GG are presented in separate graphs, panels (a) and (b), respectively. RT: reaction times

As illustrated in Figure 1a, overall mean response latencies were faster and more accurate to spatially corresponding than to noncorresponding stimuli, thus producing the expected Simon effect (*Correspondence*,* F*(1, 23): RT, *F *=* *138.03, *p *<* *0.001, *ƞ* ^2^ = 0.86; Acc, *F *=* *15.42, *p *<* *0.01, *ƞ* ^2^ = 0.40). It is also apparent in Figure 1 that overall mean response speed and accuracy did not differ between the two variations of the Taq1 polymorphism (*Group*,* F*(1, 23): RT, *F *=* *0.12, *p *=* *0.73, *ƞ* ^2^ = 0.01; Acc, *F *=* *0.07, *p *=* *0.79, *ƞ* ^2^ = 0.003) and medication states did not affect performance either (*Medication, F*(1, 23): RT, *F *=* *1.85, *p *=* *0.19, *ƞ* ^2^ = 0.07; Acc, *F *=* *0.77, *p *=* *0.39, *ƞ* ^2^ = 0.03). Figures 2 and 3 illustrate the first‐ and second‐order relations between medication, Taq1A group, and correspondence. There was a trending interaction between *Correspondence* and *Medication* (Acc, *F*(1, 23)=4.12, *p *=* *0.05, *ƞ* ^2^ = 0.15), in that patients on medication tended to be more accurate on noncorresponding trials compared to patients off medication, whereas performance on corresponding trials remained equal between medication states. None of the remaining interactions involving *Correspondence, Group,* and *Medication* were statistically significant (*p*s > 0.25).

#### 3.1.2 Response capture

**Figure 4:**
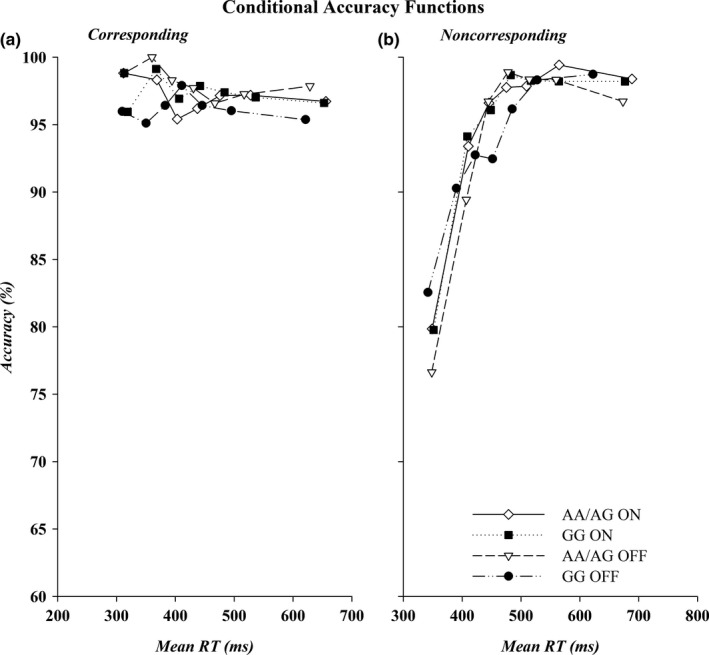
CAFs for corresponding (a) and noncorresponding trials (b) by genetic subtype. Accuracy, shown on the *y*‐axis, is plotted against mean bin RT, shown on the *x*‐axis for the fastest (Bin 1) to the slowest (Bin 7) bins. RT: reaction times

The CAFs for the AA/AG and GG subgroups of the Taq1A allele are shown in Figure 4. Most of the errors were made to noncorresponding stimuli irrespective of subgroup or medication state. The percentage of correct responses for the fastest RT bin was used for the analysis. Impulsive errors in this bin, as reflected in low accuracy rates, were higher on noncorresponding than on corresponding trials (79.7% vs. 97.40%) (*Correspondence*,* F*(1, 23) = 28.72, *p *<* *0.0001, *ƞ* ^2^ = 0.56). However, both the AA/AG and GG group were equally likely to commit fast impulsive errors (*Group*,* F*(1, 23) = 0.005, *p *=* *0.95, *ƞ* ^2^ = 0.0002), irrespective of variations in *Correspondence* or *Medication* or in their combined variation (*F*(1, 23): *Correspondence × Group*,* F *=* *0.81, *p *=* *0.38, *ƞ* ^2^ = 0.03; *Medication × Group*,* F *=* *0.81, *p *=* *0.38, *ƞ* ^2^ = 0.03; *Correspondence ×* *Medication × Group*,* F *=* *1.13, *p *=* *0.30, *ƞ* ^2^ = 0.05). Medication state did not affect impulsive errors either (*Medication*,* F*(1, 23) = 0.001, *p *=* *0.98, *ƞ* ^2^ < 0.0001).

#### 3.1.3 Interference suppression

**Figure 5:**
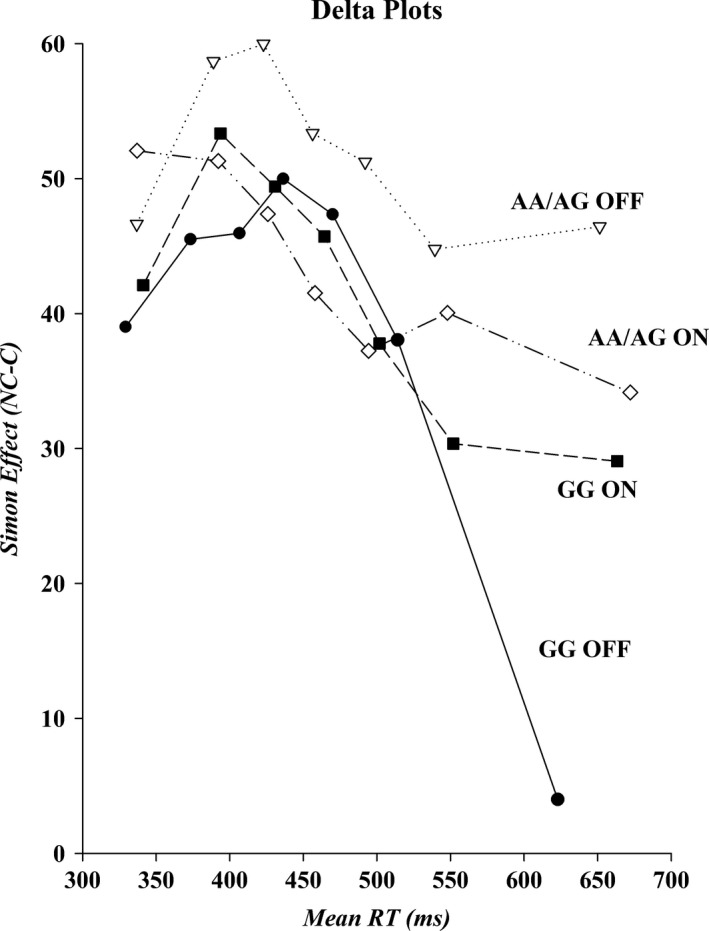
Delta plots for each genetic subtype on and off medication. The size of the Simon effect, shown on the *y*‐axis, is plotted against mean bin RT, shown on the *x*‐axis, for the fastest (Bin 1) to the slowest (Bin 7) bins. RT: reaction times

The delta plots for the AA/AG and GG subgroups of the Taq1A allele are shown in Figure 5. Analyses were restricted to the final slope and revealed no difference in steepness between AA/AG and the GG group (*Group*,* F*(1, 23) = 2.54, *p *=* *0.13, *ƞ* ^2^ = 0.10), or between medication states (*Medication*,* F*(1, 23) = 0.69, *p *=* *0.41, *ƞ* ^2^ = 0.30). However, the steepness of the slope within each group was differentially affected by medication. The AA/AG group showed a more negative‐going final slope on (−0.10) compared off medication (0.03), whereas the GG group showed the opposite pattern of a more negative‐going slope off (−0.31) versus on medication (−0.05) (*Group ×* *Medication*,* F*(1, 23) = 5.65, *p *<* *0.05, *ƞ* ^2^ = 0.20). An additional univariate ANOVA on the change scores (off minus on medication final slope values) confirmed that the group by medication effect was indeed caused by the differential effect of medication in each group (*F*(1, 23) = 5.65, *p* < 0.05, *ƞ* ^2^ = 0.20).

### 3.2 *DRD2* rs6277

#### 3.2.1 Mean RT and accuracy rates

The overall mean response latencies were faster and more accurate to spatially corresponding than to noncorresponding stimuli (*Correspondence*,* F*(1, 23): RT, *F *=* *119.64, *p *<* *0.001, *ƞ* ^2^ = 0.84; Acc, *F *=* *21.38, *p *<* *0.001, *ƞ* ^2^ = 0.48). The two variations of the polymorphism (CC/CT and TT) were not different regarding overall mean response speed and accuracy (*Group*,* F*(1, 23): RT, *F *=* *0.91, *p *=* *0.35, *ƞ* ^2^ = 0.04; Acc, *F *=* *0.64, *p *=* *0.43, *ƞ* ^2^ = 0.03) nor was there a difference between medication states (*Medication, F*(1, 23): RT, *F *=* *1.16, *p *=* *0.29, *ƞ* ^2^ = 0.05; Acc, *F *=* *0.03, *p *=* *0.87, *ƞ* ^2^ = 0.001). The difference in accuracy between corresponding and noncorresponding trials seemed smaller in patients on medication compared to when they were off medication (Acc, *Medication × Correspondence, F*(1, 23) = 6.56, *p *<* *0.05, *ƞ* ^2^ = 0.22*)* which approached significance in a paired sampled *t* test comparing the difference between corresponding and noncorresponding trials on and off medication (*t*(24) = 1.96, *p *=* *0.06, Cohen’s d = 0.33*)*. None of the remaining interactions involving *Correspondence, Group,* and *Medication* were statistically significant (*p*s > 0.12).

#### 3.2.2 Response capture

The CC/CT and TT subgroups of the rs6277 allele showed no difference in accuracy in the first bin of the reaction time distribution, (*Group*,* F*(1, 23) = 0.53, *p *=* *0.48, *ƞ* ^2^ = 0.02) and Medication did not affect accuracy either (*Medication*,* F*(1, 23) < 0.01, *p *=* *0.99, *ƞ* ^2^ < 0.001). Accuracy rates were lower on noncorresponding than on corresponding trials (80.78% vs. 97.93%) (*Correspondence*,* F*(1, 23) = 21.08, *p *<* *0.0001, *ƞ* ^2^ = 0.48). No additional interaction effects between *Group*,* Correspondence,* or *Medication* were significant, *F*s < 0.48, *p*s > 0.50.

#### 3.2.3 Interference suppression

The delta plots for the CC/CT and TT subgroups of the rs6277 allele showed no difference between the subgroups in their final delta suppression slope (*Group*,* F*(1, 23) = 1.11, *p *=* *0.30, *ƞ* ^2^ = 0.05) or between medication states (*Medication*,* F*(1, 23) = 0.35, *p *=* *0.56, *ƞ* ^2^ = 0.02). The interaction between Group and Medication was not significant either (*Group ×* *Medication*,* F*(1, 23) = 0.55, *p *=* *0.74, *ƞ* ^2^ = 0.02).

Please refer to Supporting Information Table S1 for additional data.

### 3.3 *DRD3* rs6280

#### 3.3.1 Mean RT and accuracy rates

Overall mean response latencies were faster and more accurate to spatially corresponding than to noncorresponding stimuli (*Correspondence*,* F*(1, 22): RT, *F *=* *132.05, *p *<* *0.001, *ƞ* ^2^ = 0.86; Acc, *F *=* *27.17, *p *<* *0.001, *ƞ* ^2^ = 0.55). No differences were found between the two variations of the polymorphism (CC/CT and TT) in terms of mean response speed and accuracy (*Group*,* F*(1, 22): RT, *F *=* *0.08, *p *=* *0.78, *ƞ* ^2^ = 0.004; Acc, *F *=* *0.13, *p *=* *0.72, *ƞ* ^2^ = 0.01) or between medication states (*Medication, F*(1, 22): RT, *F *=* *1.87, *p *=* *0.19, *ƞ* ^2^ = 0.08; Acc, *F *=* *0.15, *p *=* *0.70, *ƞ* ^2^ = 0.01). None of the remaining interactions involving *Correspondence, Group,* and *Medication* were statistically significant (*Fs *< 3.76, *p*s > 0.07).

#### 3.3.2 Response capture

The CC/CT and TT subgroups of the rs6280 allele showed no difference in accuracy in the first bin of the reaction time distribution (*Group*,* F*(1, 22) = 0.98, *p *=* *0.33, *ƞ* ^2^ = 0.04) and Medication did not affect accuracy either (*Medication*,* F*(1, 22) = 0.006, *p *=* *0.94, *ƞ* ^2^ < 0.001). Accuracy rates were lower on noncorresponding than on corresponding trials (80.02% vs. 97.30%) (*Correspondence*,* F*(1, 22) = 25.71, *p *<* *0.001, *ƞ* ^2^ = 0.54). No additional interaction effects between *Group*,* Correspondence,* or *Medication* were significant, *F*s < 1.09, *p*s > 0.31.

#### 3.3.3 Interference suppression

The delta plots for the CC/CT and TT subgroups of the rs6280 allele showed no difference between the subgroups in their final delta suppression slope (*Group*,* F*(1, 22) = 0.03, *p *=* *0.87, *ƞ* ^2^ = 0.001). In addition, there was no difference between medication states (*Medication*,* F*(1, 22) = 0.98, *p *=* *0.33, *ƞ* ^2^ = 0.04) or an interaction between Group and Medication (*Group ×* *Medication*,* F*(1, 22) = 0.13, *p *=* *0.72, *ƞ* ^2^ = 0.01).

Please refer to Supporting Information Table S2 for additional data.

## 4 DISCUSSION

Our results show that the Taq1A polymorphism in the *DRD2* gene (rs1800497) appears to modulate the DAAg therapeutic response on inhibitory motor control. Patients with the Taq1A polymorphism showed poorer inhibitory control of action impulses off DAAg medications and dramatic improvement on DAAg, while patients with the A2/A2 allele were much more proficient at inhibitory control off DAAg, but experienced a reduction in inhibitory control proficiency on DAAg therapy. In an interesting manner, no effect of polymorphisms or DAAg medication was seen on patients’ susceptibility to act on initial action impulses as depicted by the CAFs. Polymorphisms in rs6277 and rs6280 were not associated with differential DAAg effects on impulsive errors or on reactive inhibitory control in this study. In an important way, all of our subjects were very similar in terms of disease duration and PD severity (as measured by UPDRS motor score), so the differences seen in inhibitory control cannot be explained by disease severity.

These findings suggest that certain DRD polymorphisms may influence the direction of DA medication effects on critical cognitive control processes impaired in PD. We speculate that DAAg therapy may improve the ability to suppress impulsive action tendencies in certain patients with altered frontal–striatal D2 receptor expression. Effective inhibitory control requires a precise balance between the direct (*DRD1*) and indirect (*DRD2*) pathway, with consequences resulting from medication‐induced imbalance favoring one or the other. As the Taq1A allele results in reduced striatal DRD2 expression (8–10), PD patients with this allele may have biologic differences in the tonic activity of the indirect pathway. Reduced tonic activity of the indirect pathway results in intact fast responses (i.e., intact direct pathway) but poor motor inhibition (i.e., poor motor control when experiencing interference). We speculate that DA agonist therapy restores the balance of the direct and indirect pathway, especially in patients with reduced D2 expression (i.e., Taq1A), thereby improving action control when under conflict; conversely, patients with the TaqA2 allele have a less impaired pattern of response inhibition at baseline compared to Taq1A, but subsequently experience a decline in inhibitory control with DA agonist therapy. This is speculative but may be interesting to explore in future studies.

The results of this study have the potential to significantly contribute to our understanding of genetic variability in the DA system and how key polymorphisms may affect action control. While previous studies have also investigated the relationship between dopamine receptor polymorphisms and impulsive behaviors in Parkinson disease (Kraemmer et al., 2016; Lee et al., 2009; Vallelunga et al., 2012; Zainal Abidin et al., 2015), ours is the first to our knowledge to have examined these effects in the context of dopaminergic therapies. Understanding the complex interplay between these polymorphisms and dopaminergic medications may contribute to the highly variable effects observed in patients. Although there is not sufficient evidence to suggest that these polymorphisms determine the response to medication, it appears likely that they contribute to the variation in response that is often seen in clinical practice. The ability to predict a patient’s response to DAAg therapy on a genetic basis would significantly improve our ability to individualize treatment regimens. For instance, patients with certain polymorphisms associated with lower baseline dopaminergic activity in cortico‐striatal networks may be expected to show improvements in action control when started on DA agonists. This may translate into improvements in gait, impulsivity, and fall risk; conversely, patients with alternate polymorphisms may show a seemingly paradoxical worsening when started on DA therapy.

There are several important limitations to note in the current study. Our sample size was small, and much larger studies are clearly needed to further elucidate polymorphism effects on the cognitive and motor response to DA‐related medications. We were not able to find a differential medication response for the rs6277 or rs6280 polymorphisms in our study group, and larger sample sizes may be necessary to better determine these effects. In addition, this was a limited analysis based on a few selected polymorphisms that have been well studied in regard to addiction, self‐regulation of behavior, and other impulse control disorders. However, there are multiple additional DA receptor gene polymorphisms involving the D2 and D3 receptors, as well as the D4 receptor and the DA transporter gene, that are being identified and linked to mood, addiction, and attention‐deficit disorders as well. Further investigation is needed to determine the effects of these polymorphisms as well. A final important consideration is that we are studying a degenerating brain in the PD population. It remains to be learned exactly how these polymorphisms may affect receptor affinity in the context of neurodegeneration, which likely differs from the models based on a healthy population. In the end, it is becoming clear that genetic variability in the dopamine system plays a critical role in impulse control disorders in Parkinson’s disease and other basal ganglia disorders, and a greater understanding of these polymorphisms will have significant implications for tailoring treatment regimens in these patients.

## CONFLICT OF INTERESTS

None declared.
