# Supplementary material for: Taq1A polymorphism and medication effects on inhibitory action control in Parkinson disease
Source: Brain Behav. 2018 Jun 1;8(7):e01008. doi: 10.1002/brb3.1008 (PMC6043698; doi:10.1002/brb3.1008)
Supplement: Supplementary file 1 [file BRB3-8-e01008-s001.docx]

**Supplemental Table 1**

*DRD2 rs6277*

|  | CC/CT |  | TT |  |
| --- | --- | --- | --- | --- |
| Sample Size | OFF | ON | OFF | ON |
|  |  |  |  |  |
| RT C (ms) | 431.216 | 452.469 | 455.309 | 469.247 |
| RT NC | 471.129 | 494.181 | 510.866 | 511.924 |
| ACC C (% correct) | 96.337 | 97.088 | 98.762 | 97.514 |
| ACC NC  ACC first bin NC | 92.681  79.444 | 93.994  78.017 | 94.823  81.250 | 96.738  84.409 |
| Delta slope | -0.213 | -0.079 | -0.032 | -0.047 |
