# Supplementary material for: Taq1A polymorphism and medication effects on inhibitory action control in Parkinson disease
Source: Brain Behav. 2018 Jun 1;8(7):e01008. doi: 10.1002/brb3.1008 (PMC6043698; doi:10.1002/brb3.1008)
Supplement: Supplementary file 2 [file BRB3-8-e01008-s002.docx]

**Supplemental Table 2**

*DRD3 rs6280*

|  | CC/CT |  | TT |  |
| --- | --- | --- | --- | --- |
| Sample Size | OFF | ON | OFF | ON |
|  |  |  |  |  |
| RT C (ms) | 444.432 | 464.684 | 434.023 | 451.490 |
| RT NC | 482.885 | 503.030 | 484.292 | 497.532 |
| ACC C (% correct) | 97.464 | 97.278 | 96.541 | 97.465 |
| ACC NC  ACC first bin NC | 94.747  82.917 | 95.515  84.984 | 91.725  76.859 | 94.385  75.315 |
| Delta slope | -0.187 | -0.058 | -0.167 | -0.107 |
